# Acknowledgement to Reviewers of *Brain Sciences* in 2017

**DOI:** 10.3390/brainsci8010013

**Published:** 2018-01-10

**Authors:** Brain Sciences Editorial Office

**Affiliations:** MDPI AG, St. Alban-Anlage 66, 4052 Basel, Switzerland

Peer review is an essential part in the publication process, ensuring that *Brain Sciences* maintains high quality standards for its published papers. In 2017, a total of 160 papers were published in the journal. Thanks to the cooperation of our reviewers, the median time to first decision was 26 days and the median time to publication was 58.5 days. The editors would like to express their sincere gratitude to the following reviewers for their time and dedication in 2017:
Abhishek DesaiJohn MagnottiAdel HelmyJohn RolstonAdrian ParkeJon HennerAgnieszka FiszerJoohyung LeeAkassoglou KaterinaJosé RouillardAkiko TamasakiJosep Esteve-RomeroAlan DorvalJosephine BarnesAlan G. FinkelJoshua ParkAlan WinstonJudith S. MillerAlcy R. TorresJulie A. GriecoAlejandro López TobónJulie Gros-LouisAlessandro DidonnaJulio OrtegaAlessandro TonacciJyoti MishraAlexander U. BrandtKarolina MoustopoulouAllen L. HoKate KarelinaAmanda M. BrownKathryn M. YorkstonAmanda SeidlKathy CarterAmy MandavilleKathy MountjoyAna LedoKatsuhiko NishimoriAndré A. FentonKatsura TakanoAndre StrydomKavitha YaddanapudiAndreas LuftKaylah LalondeAndreas SchulzeKevin C. BrennanAndrej KralKevin J. BlackAndres M. LozanoKevin SchalinskieAndrew BagshawKirsten R. Müller-VahlAndrew C. TalkKylie BaileyAndrew J. SolomonLaraine WinterAndrew PostLarisa PoluektovaAndriy TemkoLarry AbelÁngel Romero MartínezLee CampbellAngela NarayanLeena MohapatraAnita Riecher-RösslerLeher SinghAnn B. RaginLeonard Verhagen MetmanAnna Fogdell-HahnLesley JonesAnna J. EsbensenLeslie SwansonAnne BeemelmannsLeyan XuAnne DezetterLili-Naz HazratiAnouk Y. J. M. SmeetsLin ZhuAnthony AkkariLindsay D. NelsonAnthony R. MawsonLisa Wise-FaberowskiAntonella ScorzielloLise EliotAntonino F. GermanoLitofsky N. ScottAntonio De TantiLora Talley WattsApit HemakomLoren MarulisArmin FuchsLori-Ann R. SacreyArthur A. VandenbarkLuca CasartelliAshraf S. GorgeyLuis R. PerazaAthanasia MouzakiLyn O’GradyAtsuhiro NakagawaLynn UlatowskiBalapal S. BasavarajappaMadelyn RayBarbara S. KoppelMarcel GiezenBarbara TettenbornMarcello CiaccioBengt KällénMarco CarotenutoBilal KhokharMarcus ChenBoris KotchoubeyMarcus GrimmBrendan RooneyMaria CabelloBrent KiousMaria ChahrourBrian ChristieMaria Chiara BuscarinuBrian Nils LundstromMaria Christina SergakiBrooke N. MacnamaraMaria HernandezBruce LyethMaria LucaBruno MeloniMariko SaitoBryan DuckhamMarion EhrichBryan HowellMarita PruessnerCalvin LiMark B. DetweilerCameron B. JeterMark R. N. KotterCamila AquinoMarkus WettsteinCarlotta Zanaboni DinaMartha MichalkiewiczCarmela BravaccioMartijn BaartCarmelo Lucio SturialeMartin Gerbert FraschCaroline JungeMartin SpülerCatherine S. Tamis-LeMondaMartina ArdizziCecilia GiuliviMARVIN R. DiazCédric GaleraMary MotzCharles A. NelsonMATTHEW ROBSONChi-Chang HuangMatthias HohmannChloe LaneMeera E. ModiChristian P. MüllerMelanie MorrisonChristian SalvatoreMelanie PinaChristine WinterMelissa BarberChristopher MillerMelissa RavenChristopher-James HarveyMichael A. Ben-AvieClaire O’CallaghanMichael D. RawlinsClare HarropMichael JohnstonClaudia MaennelMichael PerlisClaudio LiguoriMichael SchaeferClea WarburtonMichael YoongClement HamaniMichal MielcarekClodagh NolanMichel BilliardCynthia R. GrossMila VulchanovaDamian A. StanleyMohd. Farooq ShaikhDamian JenkinsMohtashem SamsamDamir JanigroMontserrat ZurrónDaniel CohenMonty SilverdaleDaniel PetersonMyra FernandesDaniel SwingleyNafisa M. JadavjiDario D. LofrumentoNathanael J. YatesDario SiniscalcoNavkiran KalsiDavid Bar-OrNawei SunDavid FacalNesha BurghardtDavid M. FergussonNiccolò MoraDavid M. SchnyerNicholas AltieriDavid P. GavinNicole OsierDavid R. ShprecherNikolaos ZiogasDavid T. DenhardtNnamdi PoleDavid W. EvansNoémie Auclair OuelletDavid WagnerNoman NaseerDavid ZieglerNora Vanegas ArroyaveDavide BarbagalloNúria Esteve-GibertDeane AikinsOlaf HaukDeAnna L. AdkinsOliver QuarrellDeborah FergusonOnintza SagredoDenise GobertOwen CarmichaelDenise SharonOzgul GokDennis W. VanePaisit PaueksakonDevanshi SethPanagiotis BamidisDevin W. McBridePaola RusminiDhanabalan MuraliPaolo CalabresiDiego Torres-RussottoPatricia BroderickDomenico De BerardisPatricia K. CoyleDomenico ServelloPatrícia MacielDominic ChengPatrizia GiannoniDora LozsadiPaul E. GreeneDouglas WeldonPaul GorczynskiDwight PiercePaul WilliamsonE. Richard StanleyPeik GustafssonEberhard UhlPeter C. Van Der EndeEdward V. QuadrosPeter E. WaisEdward VulPeter N. SteinmetzEgilius L. H. SpieringsPeter RumneyEiichi SuehiroPhilip HazellEling De BruinPhilippe SchuchtElizabeth A. StricklandPhillip TsengEmily D. RickettsPhu HoangEmily EdmondsPierre DuquetteEmily S. NicholsPierre RoubertouxEric BravermanPieter J. HoekstraEric GlasgowPranela RameshwarEric VerinQingzhong RenErik SistermansRadwa BadawyErika SkoeRakesh Kumar TiwariErwin B. MontgomeryRamon Y. BirnbaumEugene ParkRany MakaryusFabiana GeraciRasheda Arman ChowdhuryFabio CampanellaRay MeddisFabio Di DomenicoRebecca FortgangFarid RahimiRebecca ParkFelix Jimenez-RondanRemy BationFelix SeptiantoRenee HollingsworthFeng GengReto StockerFeng GuRicardo S. OsorioFeng Vankee LinRichard C. DethFernando L. ValeRichard E. MainsFilmer ChuRichard FryeFiras BannoutRichard J. YouleFlorence GressierRifai ChaiFlorence LevyRobert B. MichaelFlorien BoeleRobert G. MairFlorin DespaRobert GonzalezFrancesco BartoliRobert KotloskiFrancesco Carlo MorabitoRobert LalondeFrancesco RigoliRobert R. HanseboutFrancisca S RodriguezRobert T. MalletFrancisca S. RodriguezRocco Salvatore CalabroFranco GemignaniRodger J. ElbleFrank FlemischRodrigo NosedaFrédéric CauseretRomain Vuillefroy De SillyFrederik SteynRosella AbetiFredrik KarlssonRosemary SheehanFriedemann PaulRupsa DattaFulvio LauretaniRussell SchacharGabriel G. De La TorreRyan A. StevensonGabriela G. WernerSamuel FrankGanesh NaikSandy ShultzGarnett P. McMillanSanjay MaggirwarGary SimonSarah BaylessGayannée KediaSarah HellewellGeon Ho BahnSarah LagemanGeorg WidhalmSarah RabbittGeorge ChrousosScott R. SchroederGeorge OpieSebastian GluthGeorgios PaslakisShashi S. SeshiaGholson LyonShervin AssariGiacomo VivantiShraddha RegeGianluca SerafiniShyam SeetharamanGiordano D'UrsoSigny SheldonGiovanni AssenzaSiobhain M. O'mahonyGiri Kumar ChandakaSoledad ZarateGlen BergeronSonja ScholzGordon HarperSonya L. JakubecGregory FricchioneSophia FrangouGregory HeathStan KutcherGregory P. MuellerStéphanie Chambaron-GinhacGuennadi SaikoStephen HollandGurudutt PendyalaStephen HoneybulH. Lee SwansonStephen R. JacksonHans Van Der SteenStephen SawcerHans-Gert BernsteinSteven A. J. ChamuleauHarrison C. WalkerSteven PassmoreHasse WalumSusan J. NicolsonHeather BowlingSusan JergerHideya KodamaSusann M. Brady-KalnayHiran ThabrewSu-Youne ChangHiroaki WakimotoSven KroenerHirokazu DoiT. Dianne LangfordHolly BowenTaku HatanoHon Wah LeeTatjana AueHong JiaoTerri PogodaHovagim BakardjianThanh Dang-VuHoward ChertkowThérèse M. JayHoward PrenticeThomas HeinbockelIlya BezprozvannyThomas W. WeickertInga D. NeumannTilman HenschIngvild Saksvik-LehouillierTimothy J. CrowIsaac T. SchieferTimothy RittmanItoh YasuhiroTobias DerfussJ. Leigh LeasureTobias S. AndersenJacqueline E. ReznikTöres TheorellJaishankar BharatharajTrinidad Montero-MelendezJake NeumannTrishna PatelJames AdamsUlrich PalmJames O'CallaghanVicki L. KristmanJames R. CouchVictor RiveraJannette Rodríguez-PallaresVictor V. DyakinJared W. YoungVictoria M. LeavittJavier Ortuño-SierraVincent R. HarleyJayashri GhoshVincenzo GuidettiJean R. HughesVincenzo NataleJean-Philippe LangevinVirginia V. W. McIntoshJennifer BeerVishwanath SankarasubramanianJennifer L. LyonsWenchao ZhouJennifer RodgerWendy O. KalbergJeroen L. A. PenningsWilhelm MistiaenJérôme BrunelinWilliam D. KearnsJérôme DevyWissam DeebJerzy BodurkaWolfgang MannJessica RobinWoo-Yang KimJia ChengXiaobing YuanJie ShiXueni PanJill CrittendenYadvinder AhiJinyuan WangYang ZhangJoachim FandreyYe XiongJodi M HeapsYuan-Pin LinJoerg WischhusenYuri BozziJohanna BickYvonne HoellerJohn GruzelierZhuo XingJohn L. Woodard



